# Impact of Chemotherapy Delay on Overall Survival for AML with *IDH1/2* Mutations: A Study in Adult Chinese Patients

**DOI:** 10.1371/journal.pone.0140622

**Published:** 2015-10-14

**Authors:** Jing-Han Wang, Qi Guo, Zhi-Xin Ma, Qiu-Ling Ma, Meng-Xia Yu, Xiu-Feng Yin, Sha-Sha Lu, Hong-Qiong Xie, Yue-Hong Jiang, Dan Shen, Li-Ya Ma, Hui Shi, Wen-Juan Yu, Ye-Jiang Lou, Ying Li, Min Yang, Gai-Xiang Xu, Li-Ping Mao, Jian-Hu Li, Huan-Ping Wang, Dong-Mei Wang, Ju-Ying Wei, Hong-Yan Tong, Jian Huang, Jie Jin

**Affiliations:** 1 Department of Hematology & Institute of Hematology, The First Affiliated Hospital, Zhejiang University, Hangzhou, China; 2 Key Laboratory of Hematopoietic Malignancies, Zhejiang Province, Hangzhou, Zhejiang, PR China; 3 Department of Nephrology, The First Affiliated Hospital, Zhejiang University, Hangzhou, China; RWTH Aachen University Medical School, GERMANY

## Abstract

The effect of time from diagnosis to treatment (TDT) on overall survival of patients with acute myeloid leukemia (AML) remains obscure. Furthermore, whether chemotherapy delay impacts overall survival (OS) of patients with a special molecular subtype has not been investigated. Here, we enrolled 364 cases of AML to assess the effect of TDT on OS by fractional polynomial regression in the context of clinical parameters and genes of *FLT3*ITD, *NPM1*, *CEBPA*, *DNMT3a*, and *IDH1*/*2* mutations. Results of the current study show *IDH1/2* mutations are associated with older age, M0 morphology, an intermediate cytogenetic risk group, and *NPM1* mutations. TDT associates with OS for AML patients in a nonlinear pattern with a J shape. Moreover, adverse effect of delayed treatment on OS was observed in patients with *IDH1/2* mutations, but not in those with *IDH1/2* wildtype. Therefore, initiating chemotherapy as soon as possible after diagnosis might be a potential strategy to improve OS in AML patients with *IDH1/2* mutations.

## Introduction

Acute myeloid leukemia (AML) is a heterogeneous group of hematologic malignancies characterized by the rapid growth of leukemia cells requiring immediate therapeutic intervention. Currently, the clinical of outcome of patients is poor after treatment with conventional chemotherapy. However, recent progression in AML treatment is focused, in part, on the development of targeting therapies. Therefore, there is a need to evaluate AML using cytogenetic and molecular analyses accurately and extensively immediately after diagnosis to utilize targeted therapies earlier. It is well known the morphologic diagnosis of AML can be easily detected after only a few hours, but cytogenetic and/or molecular analyses results can require one week or longer. Thus, waiting on laboratory test results delays treatment. It is worth noting that it will, more often than not, take time to transfer patients from first suspicion of leukemia to final diagnosis at an experienced hematologic center. Therefore, whether the time from diagnosis to treatment (TDT) impacts overall survival is becoming one of the most important clinical issues. To the best of our knowledge, there are three retrospective studies in western populations which demonstrate this question. In the first study, clinical outcome was worsened after a chemotherapy delay of 5 days for younger AML patients, but not older patients[1]. In contrast, the second report suggests TDT has no impact on the outcome of patients with AML[2]. The third study suggests delaying intensive treatment has an adverse impact on the prognosis in both younger and older AML patients in a Danish population-based cohort[3]. In summary, the prognostic significance of TDT in AML patients remains controversial. It is worth noting several gene mutations such as *IDH1/2* mutations are closely correlated to older age[4], and age was regarded as an interactive factor for the prognosis of TDT in a previous report[1]. However, these studies did not take into account the effect of gene mutations when investigating the prognostic impact of TDT on overall survival. So we hypothesized that TDT can be modified by well-established predictors such as gene mutations, and correspondingly designed a retrospective study to address this issue. We found that delayed TDT was an adverse predictor for overall survival occurring in patients with *IDH1/2* mutations but not in those with *IDH1/2* wild type. This result introduces a new way for clinicians to improve outcomes by the tailored therapy as soon as diagnosis occurs for *IDH1/2* mutant cases.

## Materials and Methods

### Patients

Clinical data were abstracted from medical records of AML patients in Zhejiang Institute of Hematology (ZIH), which is one of the hematologic centers in China. Between March 2008 and June 2013, 364 patients with detailed diagnoses and treatment information were enrolled in this study. WHO classification, conventional cytogenetic banding assay, and molecular analyses were performed as previously described in AML diagnosis[5]. Cytogenetic groups of patients were classified as favorable, intermediate, and unfavorable risk according to the NCCN guideline[6]. Favorable subgroups included t(8;21)/*AML1-ETO* and inv16/*CBFβ-MYH11*; adverse consisted of t(9;22), inv(3)/t(3;3), -5, -7, del(5q), del(7p), 11q23 and complex translocations; intermediate subtype contained cytogenetically normal and AML with other cytogenetic abnormalities. Patients were treated with standard anthracycline and cytarabine or HAA (homoharringtonine combined with cytarabine and aclarubicin) protocol for induction chemotherapy as previous reported[7, 8]. In the consolidation therapy, younger patients were treated with a high-dose cytarabine-based chemotherapy[7]. The chemotherapy consolidation for elderly patients was decided by the physicians in an individualized manner, as described previously[7]. No patient in our study received allogeneic transplantation. Patients with secondary AML or acute promyelocytic leukemia were excluded. We also excluded patients who did not receive chemotherapy beyond 45 days after disease diagnosis. All of the subjects were well-informed about the study and provided written informed consent to participate in the study. The study was approved by the Institutional Review boards of the First Affiliated Hospital of Zhejiang University.

### Cytogenetic and Gene mutation analysis

The BM samples of de novo AML patients were studied mostly by R-banding analysis. Chromosomal abnormalities were described according to the International System for Human Cytogenetic Nomenclature [9]. DNA and RNA samples of AML patients were obtained from mononuclear cells isolated by Ficoll gradient centrifugation from bone marrow samples at primary diagnosis. Gene mutations of *NPM1*, *FLT3*ITD, *CEBPA*, and *DNMT3a* were analyzed by whole-gene sequencing as previously described [10]. RNA samples were used to determine *PMLRARA*, *AML1ETO*, and *CBFβMYH11* fusion genes by reverse transcription polymerase chain reaction (RT-PCR). *IDH1* and *IDH2* mutations were determined by cDNA amplifications. The PCR primers used were forward, 5'-TCCCTACGTGGAATTGGATCTAC-3' and reverse, 5'-TCACCTTTTGGGTTCCGTCA-3' for *IDH1* and forward, 5'-TGGCCACCCAGAAGTACAGTG-3' and reverse, 5'-TGGCATACTGGAAGCAGCTG-3' for *IDH2*. PCR reactions were performed in a total volume of 25μl containing of 1μl of 100 ng/μl sample cDNA, 12.5μl of 2×PCR Mix, 1μl of 0.5μM of each primer, and 10.5μl of ddH2O. All PCR products were directly sequenced with both forward and reverse primers to ensure quality. All sequence data were read using Chromas version 2.22 software.

### Definition of clinical end points and statistical analysis

Patient characteristics were summarized using descriptive statistics, which included frequency counts, median, and inter-quartile. TDT was defined as the number of days between the first bone marrow aspirate and chemotherapy initiation. The relationship between TDT and patient characteristics was evaluated by the nonparametric test. The primary end point of the study was overall survival (OS). OS was measured as time from disease diagnosis to death from any cause, or censoring for patients alive at their last known date of contact.

Initially, the effect of TDT on OS was evaluated as a categorical variable using the log-rank test in the Kaplan-Meier (KM) survival model. Survival curves suggested the relationship between TDT and OS was nonlinear. To avoid loss of information and a reduction in power introduced by the categorical variable of TDT, we further investigated the nonlinear relationship between TDT and OS using fractional polynomial (FP) algorithm, of which kept TDT as a continuous variable in Cox regression model. FP models have been proposed for investigating main effects of predictors for possible non-linearity[11]. The proportional-hazards assumption was checked for each variable before fitting Cox models. For the binary or categorical predictors, the effects on OS were evaluated by log-rank test in Kaplan–Meier (KM) survival analysis. Variables with a p-value less than 0.2 were selected as adjustment covariates into the multivariable analyses. To evaluate the interaction between TDT and covariates, multivariate fractional polynomial (MFP) interaction analysis including MFPIgen and MFPI were conducted. MFPI firstly used MFP to conduct multivariate analysis and then tested for significant interaction terms using a deviance difference test[4, 12]. MFPIgen was an extension to MFPI for modeling continuous-by-continuous interactions in a multivariable context[12]. The interaction was also validated by log-rank test in KM survival analysis. Finally, we performed the sensitivity analysis of interactions to adjustment models with different covariates. All statistical analyses were conducted with STATA Statistical Software (Version 11; College Station, TX) and R statistic packages, version 2.15.0 (www.r-project.org). The two-sided level of significance was set at p-value less than 0.05.

## Results

### Characteristics of patients with *IDH1/2* mutations

Of 364 patients, 85 (23%) had *IDH1/2* mutations. *IDH1* mutations were detected in 39 (11%) patients and *IDH2* mutations were detected in 48 (13%) patients. Only 2 patients had both *IDH1* and *IDH2* mutations. As *IDH1* and *IDH2* mutations were mutually exclusive and appeared to have same biologic functions, we examined the clinical features of *IDH1* and *IDH2* mutations as a collective group. Clinical characteristics of patients with *IDH1/2* mutations are described in Table 1. *IDH1/2* mutations were associated with older age (median, 57 years vs. 47 years, P<0.001), more frequent in M0 morphology (P<0.001), cytogenetic intermediate risk group (P = 0.04), and higher in the frequency of *NPM1* mutations (P = 0.02). We also found patients with *IDH1/2* mutations tended to show higher frequency of *DNMT3a* mutations (18% vs. 10%, P = 0.07). However, *IDH1/2* mutations were mutually exclusive with double allele *CEBPA* mutations (2% vs. 9%, P = 0.04). There was no statistically significant correlation between *IDH1/2* mutations and other variables including percent blast, white blood cell counts, sex, and *FLT3*ITD (Table 1).

**Table 1:** Comparisons of clinical and molecular features in AML patients with and without *IDH1/2* mutations.

Variable	*IDH1/2* WT	*IDH1/2* mutations	P-value
**Age, median(range), yrs**	47(14,82)	57(15,78)	<0.001
**Percent blast, median(range)**	65(20,98)	73(20,97)	0.15
**WBC, median(range),10^9/L**	11(0.2,487)	14(0.4,262)	0.50
**Female, n(%)**	114(41)	32(38)	0.60
**FAB classification, n(%)**			<0.001
**M0**	15(5)	18(21)	
**M1**	19(7)	7(8)	
**M2**	93(33)	29(34)	
**M4**	56(20)	6(7)	
**M5**	90(32)	22(26)	
**M6**	6(2)	3(4)	
**Cytogenetic subtype, n(%)**			0.04
**Favorable**	30(11)	2(2)	
**Intermediate**	211(76)	70(82)	
**Adverse**	38(14)	13(15)	
**Gene mutations, n(%)**			
***FLT3*ITD**	41(15)	16(19)	0.36
***NPM1***	60(22)	29(34)	0.02
***CEBPA*** ^***DM***^	25(9)	2(2)	0.04
***DNMT3a***	29(10)	15(18)	0.07

WT: wild type; WBC: white blood cell counts; FAB: French-America-British; DM: double-allele.

### Association of TDT with clinical characteristics

In our cohort of AML patients, we found that patients had a median TDT of 5 days, inter-quartile of 3 to 9 days, and the range of 1 to 45 days, respectively. Interestingly, we found the variables significantly associated with longer TDT were older age (P = 0.001), FAB M0 classification (P = 0.009), *IDH1/2* mutation (P = 0.002), below median of blast (P = 0.001), and lower levels of white blood cell counts (P = 0.001) (Table 2). There was no significant correlation of TDT and variables including sex, cytogenetic risk group, and genes of *FLT3*ITD, *NPM1*, *CEBPA*, and *DNMT3a* mutations.

**Table 2:** Comparisons of TDT according to patients’ characteristics.

Variable	Number (%)	TDT[median(IQR),days]	P-value
**Age (years)**			<0.001
**< 60**	276(76)	5(3,8)	
**> = 60**	88(24)	7(4,13)	
**Sex**			0.64
**Male**	218(60)	6(3,9)	
**Female**	146(40)	5(3,9)	
**FAB classification**			0.009
**M0**	33(9)	7(5,8)	
**M1**	26(7)	6(4,8)	
**M2**	122(34)	6(3,12)	
**M4**	62(17)	5(1,8)	
**M5**	112(31)	4(2,7)	
**M6**	9(3)	6(4,13)	
**Cytogenetic subtype**			0.33
**Favorable**	32(9)	6(3,10)	
**Intermediate**	281 (77)	5(3,9)	
**Adverse**	51(14)	5(2,8)	
**Percent blast**			0.001
**Above median**	182(50)	5(2,7)	
**Below median**	182(50)	7(3,12)	
**WBC, (10^9/L)**			0.001
**Above median**	182(50)	4(2,7)	
**Below median**	182(50)	6(4,10)	
**Gene mutations**			
***FLT3*ITD**			0.61
**Mutant**	57(16)	5(3,8)	
**Wildtype**	307(84)	5(3,9)	
***NPM1***			0.87
**Mutant**	57(16)	5(3,9)	
**Wildtype**	307(84)	5(3,9)	
***CEBPA***			0.20
**DM Mutant**	27(7)	6(4,9)	
**Wildtype**	337(93)	5(3,9)	
***DNMT3a***			0.68
**Mutant**	44(12)	5(3,8)	
**Wildtype**	320(88)	5(3,9)	
***IDH1/2***			0.002
**Mutant**	85(23)	7(4,14)	
**Wildtype**	279(77)	5(3,8)	

IQR: inter-quartile; FAB: French-America-British; WBC: white blood cell counts. DM: double-allele.

### TDT associated with overall survival in AML patients

**Fig 1:**
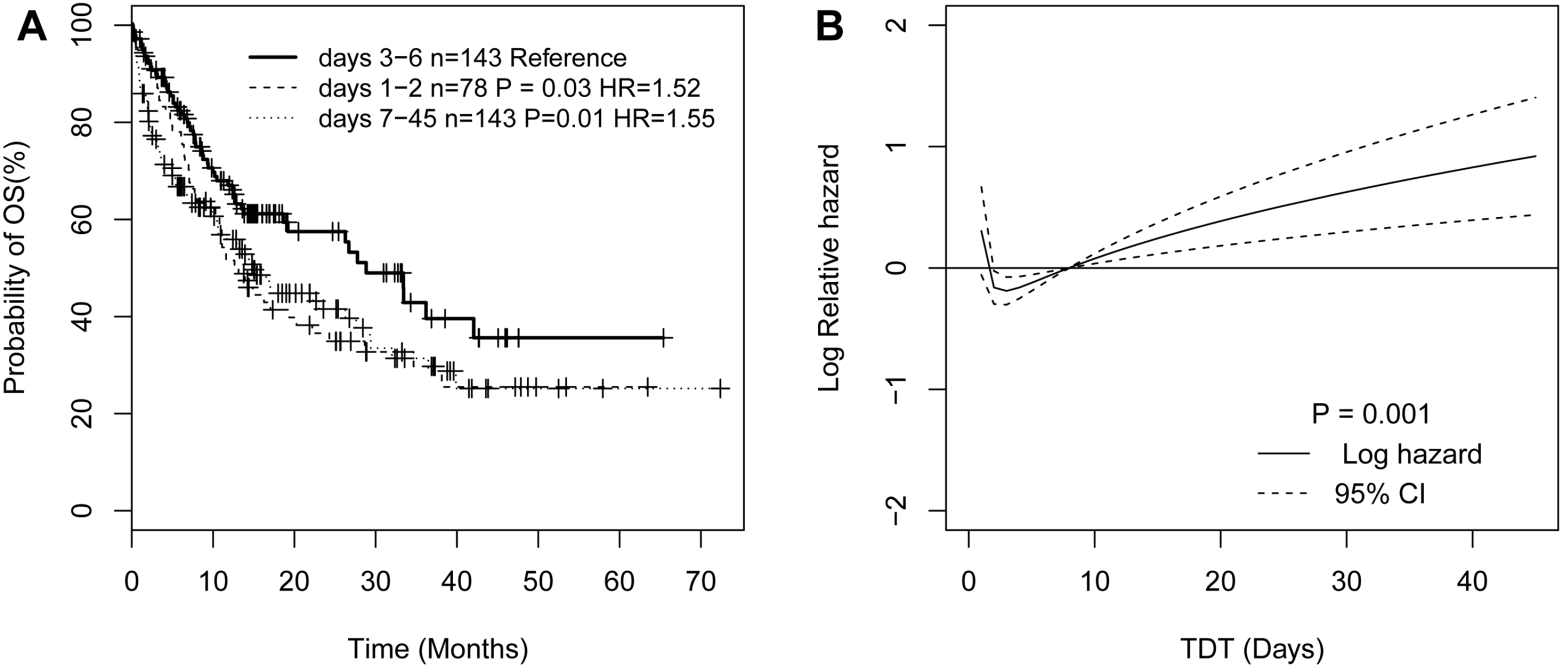
The plots illustrating the nonlinear relationship between TDT and overall survival. Kaplan–Meier survival curves illustrating the effect of TDT as a categorical variable on overall survival (A). The plot of the estimated log hazard in mortality using fractional polynomial algorithm together with its 95% confidence (B).

In order to better understand the relationship between TDT and OS, we define the TDT categories according to the workflow of our clinical laboratory test. Generally, the results of morphologic, immunologic, cytogenetic, and/or molecular (MICM) diagnosis are required and available to AML-diagnosis in 5 or 6 days. In fact, MICM diagnosis of AML will be made for all patients within 12 days in our clinical center. Therefore, we divided patients into two groups: the first group consisted of 303 (83%) patients with TDT less than 13 days, and the second group included 61 (17%) patients with TDT beyond 13 days. We further classified patients into six subgroups based on the adjacent TDT interval: days 1–2 group (78 cases), days 3–4 group (72 cases), days 5–6 group (71 cases), days 7–8 group (49 cases), days 9–10 group (21 cases), and days 11–12 group (12 cases), respectively. We then conducted survival analysis using group 13–45 days as a reference, because the majority of this group characterized poor performance status and severe infection and was unfit for chemotherapy after disease diagnosis. As a result, days 3–4 group (P = 0.02), and days 5–6 group (P = 0.048) had favorable OS, whereas, the survival of the other subgroups (days 1–2 group, days 7–8 group, days 9–10 group, days 11–12 group) was similar to the reference group (S1 Table and S1 Fig). Therefore, we refine patients into three groups according to the similar p-values: days 1–2 group, days 3–6 group, and days 7–45 group, respectively. The non-linear relationship between TDT and OS is more apparent: days 1–2 group (P = 0.03, HR = 1.52) and days 7–45 group (P = 0.01, HR = 1.55) had an adverse overall survival respectively compared to days 3–6 group (Fig 1A).

To demonstrate the detailed nonlinear pattern between OS and TDT, we further conducted the FP regression in the Cox model. The best FP fitting function for TDT included the terms TDTI^-2^ and TDTI^0.5^, where TDTI = TDT/10, which was significantly associated with overall survival (P = 0.001). As shown in Fig 1B, the estimated survival curves of TDT for OS were a J shape, with a nadir from approximately day 2 to day 7, and the fall of TDT from 0 to 3 days and then increase again with the increase of the TDT from 8 days or more. We also found age, white blood cell count, cytogenetic subtypes, genes mutations of *FLT3*ITD, *CEBPA*, *DNMT3a*, and *IDH1/2* were significantly associated with OS (S1 Table). In addition, we found that FP terms of TDT (TDTI^-2^ and TDTI^0.5^, where TDTI = TDT/10) were the independent predictive factors after adjusting for WBC (continuous), age (continuous), cytogenetic subtypes, genes of *FLT3*ITD, *NPM1*, *CEBPA* double allele, *DNMT3a*, and *IDH1/2* mutations (S2 Table).

### Chemotherapy delay confers poor overall survival in AML patients with *IDH1/2* mutations

**Fig 2:**
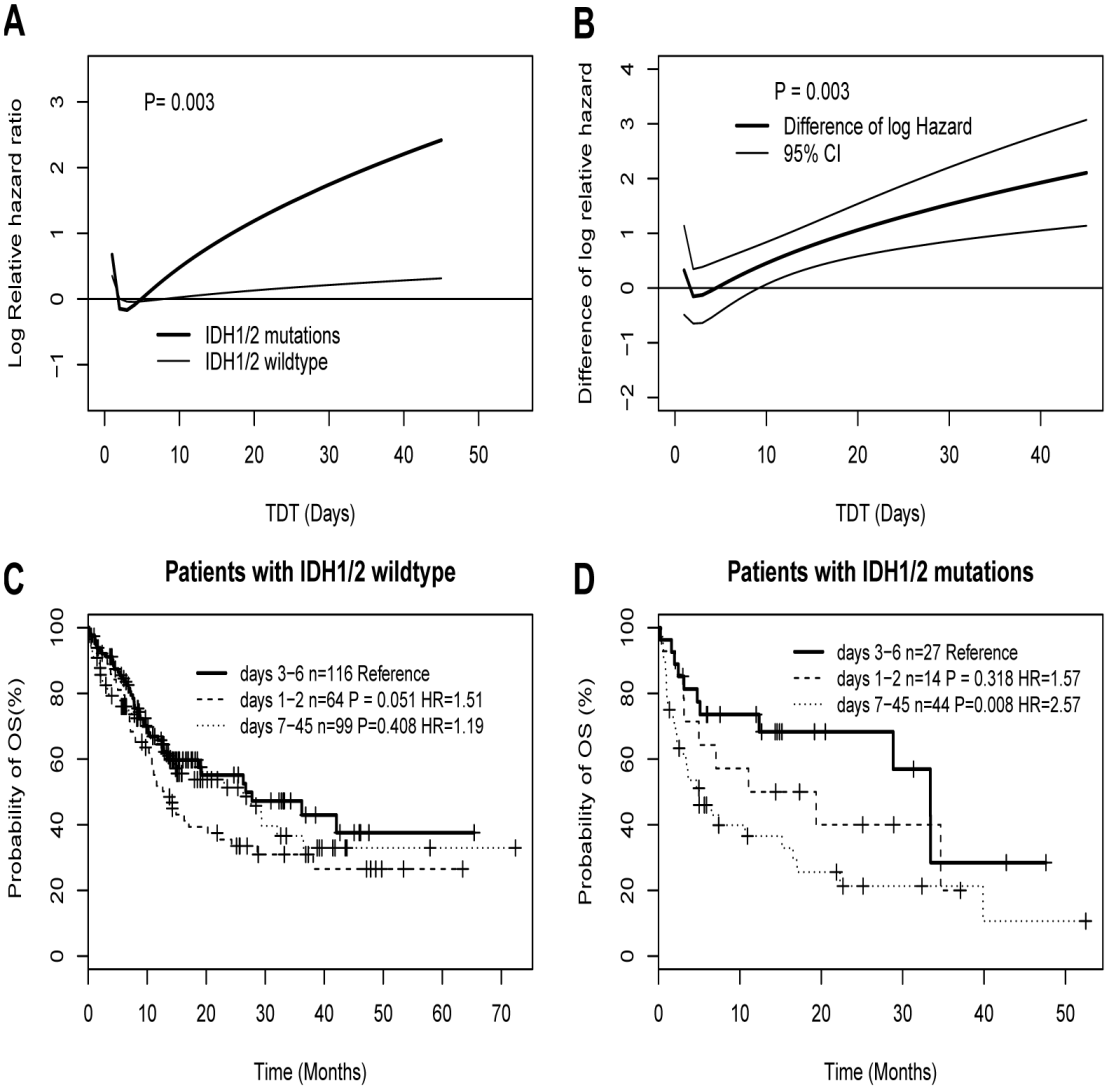
The plots illustrating the interaction between *IDH1/2* mutations status and TDT. Log hazard in mortality for AML patients by *IDH1/2* mutation status (A), and estimated difference of log hazard in mortality between *IDH1/2* mutations and wildtype together with its 95% confidence band (B). Figure C shows that for patients with *IDH1/2* wildtype the survival rates decrease in a similar fashion, whereas Figure D shows *IDH1/2* mutant patients with a TDT of 7 days or more have a lower survival rate. MFPIgen function (A) and MFPI function (B) were respectively estimated the interaction effects. Kaplan–Meier survival curves (C and D) illustrating the interaction between TDT and *IDH1/2* mutations.

In order to further explore whether the relationship between OS and TDT was modified by the clinical and molecular factors, we considered the interactive effects between TDT and covariates using MFPIgen models. As shown in Fig 2A, we found there was an interaction between TDT and *IDH1/2* mutations in survival analysis (p-value of interaction = 0.003 in MFPIgen algorithm). As shown in Fig 2B, based on the difference of log hazard ratio in MFPI algorithm, those with a TDT of 7 days or more do not seem to benefit in survival rates from *IDH1/2* mutations (P = 0.003). The survival curves of Fig 2C further illustrates there are no significance difference in the three groups among patients with *IDH1/2* wildtype (days 1–2 group vs. days 3–6 group: P = 0.051; days 7–45 vs. days 3–6 group: P = 0.408). However, for patients with *IDH1/2* mutations (Fig 2D), days 7–45 group had a significant adverse OS compared to days 3–6 group (P = 0.008, HR = 2.57), and no significant survival difference was observed between days 1–2 group and days 3–6 group (P = 0.318, HR = 1.57). In patients with *IDH1/2* mutations, FAB subgroups between patients with and without delayed treatment beyond 7 days are randomly distributed (S3 Table). Furthermore, the adverse effect of chemotherapy delay beyond 7 days remains significant in the multivariate analysis after adjusting the most well-established predictors such as age, WBC, cytogenetic risk groups, and genes of *FLT3*ITD, *NPM1*, *CEBPA*, and *DNMT3a* mutations (Table 3). In addition, these interactions were also observed by sensitivity analyses using different adjusted confounding factors such as clinical variables including age (continuous), WBC (continuous), and/or molecular variables like cytogenetic risk group and genes of *FLT3*ITD, *NPM1*, *CEBPA*, *DNMT3a*, and *IDH1/2* mutations in different adjusted models, respectively (S2 Fig). It is worth noting that TDT also remains significant in the context of clinical and molecular factors (S4 Table). However, we did not found other interaction between TDT and the other potential predictors including age, WBC, percent blast, cytogenetic subtypes, and genes of *FLT3*ITD, *CEBPA*, *NPM1* and *DNMT3a* mutations (S3 Fig).

**Table 3:** Impact of chemotherapy delay beyond 7 days by *IDH1/2* mutations status in Cox multivariate regression analysis.

Variables	HR(95%CI)	P value
**Age**	1.02(1.004,1.03)	0.008
**WBC**	1.003(1.001,1.005)	0.001
***DNMT3a***	1.75(1.16,2.65)	0.008
***NPM1***	0.81(0.56,1.18)	0.278
***FLT3*ITD**	1.15(0.73,1.82)	0.537
***CEBPA*** ^***DM***^	0.11(0.03,0.44)	0.002
**Cytogenetic risk group**		
**Intermediate vs. Favorable**	2.21(1.14,4.29)	0.020
**Adverse vs. Favorable**	2.71(1.24,5.94)	0.013
**IDHm&Day7 vs. IDHm&Day6**	2.06(1.16,3.65)	0.014
**IDHw&Day6 vs. IDHm&Day6**	1.17(0.71,1.91)	0.533
**IDHw&Day7 vs. IDHm&Day6**	1.50(0.88,2.57)	0.140

WBC: white blood cell counts. DM: double-allele. IDHm&Day6: *IDH1/2* mutations and treatment within 6 days; IDHm&Day7: *IDH1/2* mutations and treatment delay 7 days or more; IDHw&Day6: *IDH1/2* wildtype and treatment within 6 days; IDHw&Day7: *IDH1/2* wildtype and treatment delay 7 days or more.

## Discussion

In this study, we found the frequency of *IDH1/2* mutations of AML patients was 23%, which is similar to most previous reports[13, 14]. In the relationship with clinical characteristics, *IDH1/2* mutations were associated with older age, intermediate risk of cytogenetic groups, and *NPM1* mutations, whereas *IDH1/2* mutations were mutually exclusive with *CEBPA* mutations. These results are also consistent with previous reports[15]. Because *IDH1* and *IDH2* mutations were mutually exclusive and appear to have identical functions[16], we examined the clinical impact of *IDH1*and *IDH2* mutations as a whole group. Interestingly, patients with *IDH1/2* mutations had longer TDT. The reason could be that *IDH1/2* mutations occur more common in FAB M0, which is required to distinguish from acute lymphoblast leukemia in morphologic diagnosis. As a result, it will take time to transfer patients with suspicion of M0 to the experienced hematologic center and wait for explicit molecular diagnosis before chemotherapy. Additionally, *IDH1/2* mutations occurred more common in elderly patients. Most of the elderly patients were chosen to be transferred to the experienced hematologic center in fear of complication after chemotherapy. Thus, these factors contribute to treatment delay.

Notably, whether treatment delay impacts overall survival in AML patients is still under investigated. A report from a Danish population indicated treatment can probably be delayed for up to 10 days without affecting the prognosis in de novo AML[3]. However, another report from a US study of 1,317 AML patients suggested delaying treatment beyond 5 days resulted in inferior survival in younger patients[1]. According to the above reports, the prognosis of TDT might be affected by the different cut-off values. To our knowledge, there is no consensus for TDT categories in survival analysis. Even though unexpected delay in the laboratory test may occur, MICM diagnosis of AML will be available for all patients within 12 days in our clinical center. If patients obtained chemotherapy after 13 days, the reason cannot be simply explained by waiting for laboratory results. The major reason patients are unfit for chemotherapy after disease diagnosis may be either severe infection or poor performance status in our routine AML management. Unfortunately, we cannot obtain all detailed information of chemotherapy delay after 13 days due to the retrospective study design. However, we can infer patients who obtained chemotherapy delay beyond 13 days must have poor survival. Therefore, we divide patients into seven groups according to the adjacent TDT intervals and took patients with chemotherapy delay 13 days or more as a reference group when we conduct KM survival analysis. The results show patients with chemotherapy delay 3 to 6 days have favorable outcomes and other patients have similar poor survival rates (S1 Fig).We further refine these patients into 3 groups according to the similar p-values. The nonlinear relationship between TDT and OS is more apparent in patients classified into the 3 subgroups (Fig 1A). Secondly, we further validated this result by survival analysis using fractional polynomial model whereas TDT was considered as a continuous variable with the power (-2, 0.5) transformation (Fig 1B). Finally, we analyzed whether the relationship between OS and TDT was confounded by others factors. In multivariate analysis, we found treatment delay was an independent predictor for overall survival in AML patients (S2 Table). This result is similar to the Danish study that TDT was a strong predictor for AML patients[3]. In contrast, a French single-center study indicated that there was no association between TDT and survival[2]. These discordant results may be due to differences in the patient populations or difference in statistical methodologies for adjusting the confounders.

In order to better understand the effect of TDT on OS in patients with the special molecular subtype, we respectively explored the interactive effect between TDT and covariates. Notably, we found there was a statistically significant interaction between TDT and *IDH1/2* mutation status (Fig 2A and 2B, P = 0.003). In Fig 2C and 2D among patients with *IDH1/2* wildtype, the survival rates decrease in a similar fashion, whereas patients with *IDH1/2* mutations appear to have a poor survival rate in those with a TDT of 7 days or more. This independent adverse effect of a chemotherapy delay of 7 days in patients with *IDH1/2* mutations was also confirmed by the multivariate analysis (Table 3). This result indicated TDT was a factor predicting for poor OS in patients with *IDH1/2* mutations but not in those with *IDH1/2* wild type. To reduce the chance of over-fitting and of incorrectly identifying interactions, we performed the sensitivity analyses by selecting different confounding factors into different adjusted models. Interestingly, when we performed the multivariate analysis in the context of clinical and molecular factors but not the interaction terms, a significantly statistical association between TDT and OS was observed (S2 Table). Furthermore, when we added the interaction terms into the above models, we found the interaction terms and TDT still remained in the different complex models adjusting clinical, molecular, and clinical and molecular factors, respectively (S2 Fig and S4 Table). These results indicated that TDT is not a poor predictor, and the interaction between TDT and *IDH1/2* mutations should not be artificial. In addition, we did not find a significant interaction between TDT and other covariates including age, WBC, percent blast, cytogenetic risk group, genes of *FLT3*ITD, *NPM1*, double allele *CEBPA*, and *DNMT3a* mutations (S3 Fig). However, Bertoli reported that there was an interaction between TDT and age as well as WBC[2].The discrepancy may be partly explained by the different included and excluded criteria and population in the different study. We also found that several well-established factors including age, WBC, cytogenetic subtypes and gene mutations of *CEBPA*, *DNMT3a*, *FLT3*ITD, and *IDH1/2* are associated with OS in a univariate analysis (S1 Table). In multivariate analysis, several well-established prognostic factors such as age, WBC, cytogenetic subtypes and gene mutations of *CEBPA* as well as *DNMT3a* were still significantly associated with OS (Table 3). However, *FLT3*ITD and *NPM1* mutations were not associated with OS in the multivariate models. The possible reason may be that the lower prevalence of these genes mutations in Chinese AML patients[5]. These results are similar to previous reports in Chinese AML patients using a large sample size[5, 7].

However, caution needs to be practiced in interpreting our results. Firstly, our results were based on the data from the single center and were not validated by another cohort of patients. Secondly, because the study was a retrospective design, the absence of explicit data was limiting in determining the exact reason why some patients received chemotherapy delay. Finally, the sample size of our patients was not large enough to evaluate the prognosis of TDT in each subgroup of patients with each special gene mutations, so additional results are required for further validation using a larger population.

Taken together, chemotherapy delay would cause an adverse effect in AML patients. We demonstrate for the first time chemotherapy delay in AML patients with *IDH1/2* mutations beyond 7 days may have an adverse effect on overall survival. However, our results are based on a retrospective cohort in a single center and required further validation by studying an independent cohort in the future.

## Supporting Information

S1 Fig Kaplan-Meier curves for patients with TDT as a multiple categorical variable. (PDF)

S2 Fig The plots illustrating the interaction between *IDH1/2* mutations status and TDT after adjusting clinical and/or molecular features. Log hazard in mortality for AML patients by *IDH1/2* mutation status after adjusting clinical variables such as age (continuous), WBC (continuous) (A). Log hazard in mortality for AML patients by *IDH1/2* mutation status after adjusting molecular variables like cytogenetic subtypes and genes mutations of *FLT3*ITD, *CEBPA*, *DNMT3a*, *NPM1* and *IDH1/2* in MFPIgen models(B). Log hazard in mortality for AML patients by *IDH1/2* mutation status after adjusting clinical and molecular variables(C).
(PDF)

S3 Fig The plots illustrating the interactive effect between the predictors and TDT on overall survival. There were no interactive effect between TDT and covariates including age (A), WBC (B), percent blast (C), cytogenetic subtypes (D), and genes mutation of *FLT3*ITD (E), *NPM1* (F), *CEBPA* (G) and *DNMT3a* (H). MFPIgen functions were used to estimate the significance of interaction between the predictors and TDT using fractional polynomial transformation with the powers (-2,0.5).
(PDF)

S1 Table Univariate analysis for overall survival in AML patients. (DOCX)

S2 Table Multivariate analysis for overall survival in AML patients. (DOCX)

S3 Table The distribution of FAB subgroups in patients with *IDH1/2* mutations by 7 days of delayed treatment. (DOCX)

S4 Table Results of multivariate analysis comparing the interaction between TDT and *IDH1/2* mutations on overall survival. (DOCX)

S1 Dataset The individual patient data. OS, overall survival; Status, 0 = alive, 1 = dead; TDT, times from diagnosis to treatment (days); TDTmc, 1 = days 1–2, 2 = days 3–4, 3 = days 5–6; 4 = days 7–8, 5 = days 9–10, 6 = days 11–12, 7 = days 13–45; TDT3C, 0 = days 2–6, 1 = days 1–2, 2 = days 7–45; WBC: white blood cell counts; IDHm&Day6,0 = *IDH1/2* mutations and treatment within 6 days,1 = *IDH1/2* mutations and treatment delay 7 days or more, 2 = *IDH1/2* wildtype and treatment within 6 days, 3 = *IDH1/2* wildtype and treatment delay 7 days or more; diagnosis,1 = M1, 2 = M2, 4 = M4, 5 = M5, 6 = M6; Cytogenetics, 1 = favorable, 2 = intermediate, 3 = poor; IDH1/2, *IDH1* and *IDH2* mutations status; DNMT3A, *DNMT3A* mutations status; NPM1, *NPM1* mutations status; *FLT3*ITD, 1 = postive, 0 = negative; CEBPMDM, double allele *CEBPA* mutations status.
(TXT)
