# Supplementary figures and images for: Impact of Chemotherapy Delay on Overall Survival for AML with IDH1/2 Mutations: A Study in Adult Chinese Patients
Source: PLoS One. 2015 Oct 14;10(10):e0140622. doi: 10.1371/journal.pone.0140622 (PMC4605653; doi:10.1371/journal.pone.0140622)

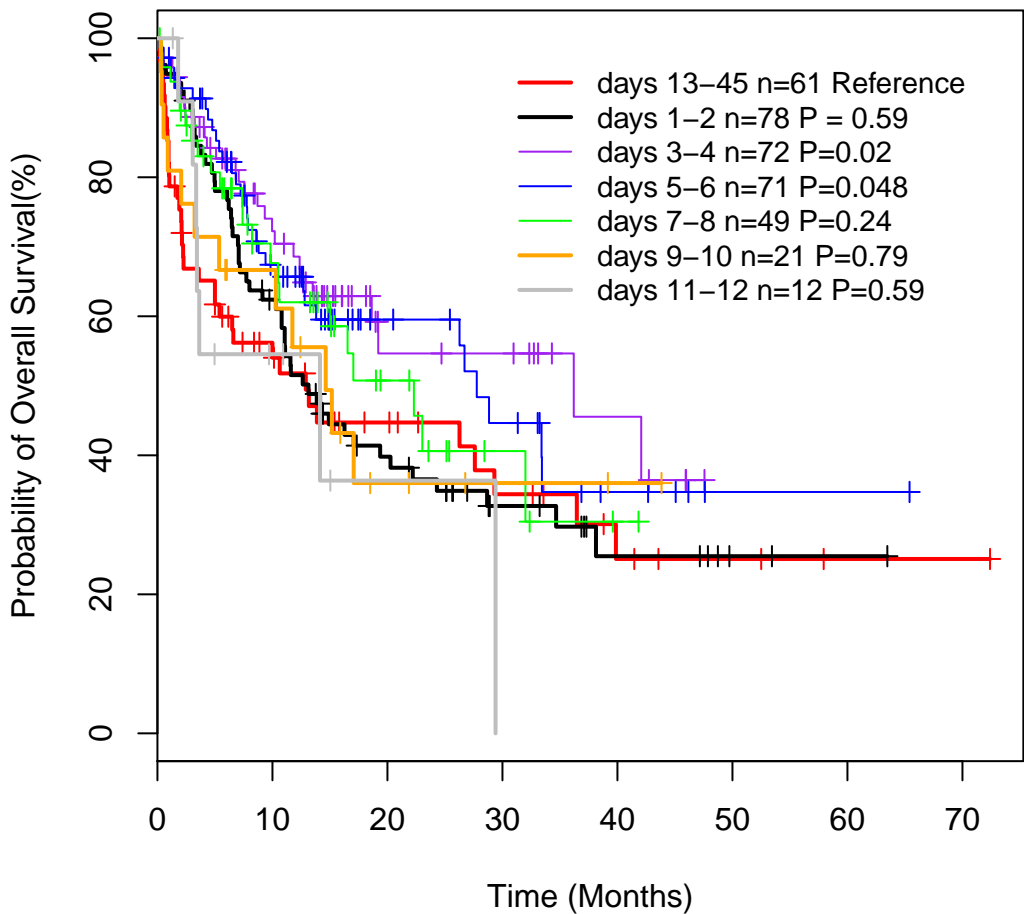

Supplement: S1 Fig — (PDF) [file pone.0140622.s001.pdf]

**A**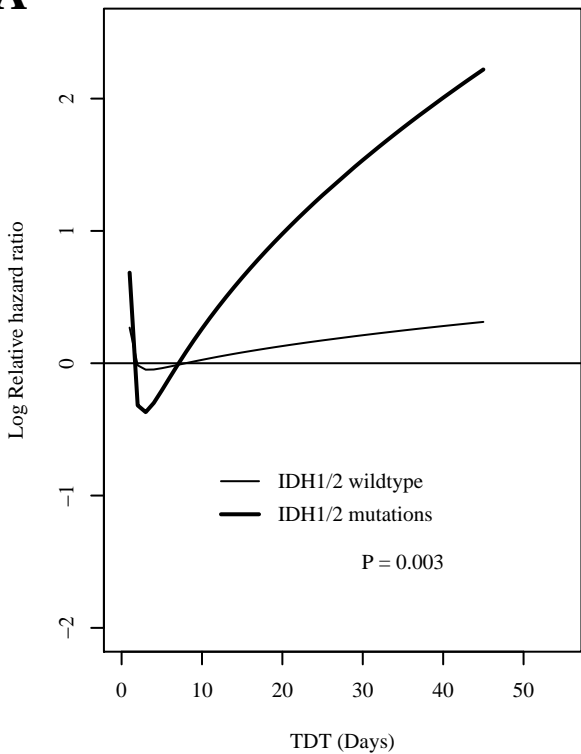**B**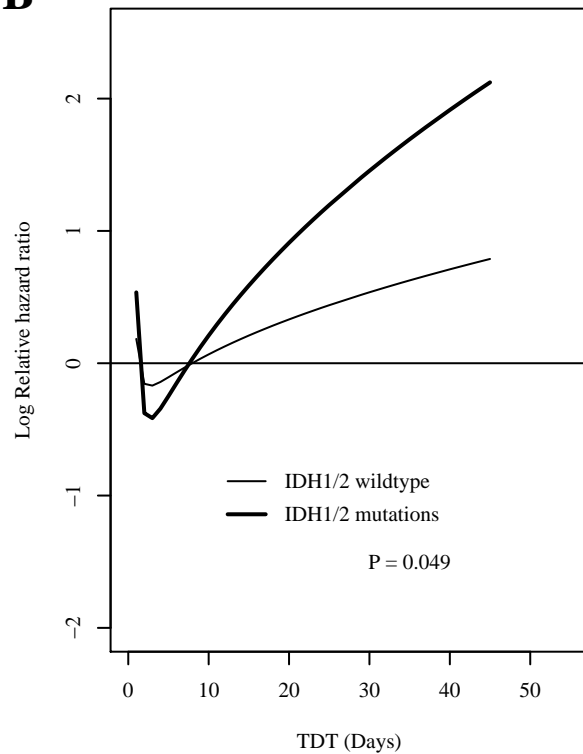**C**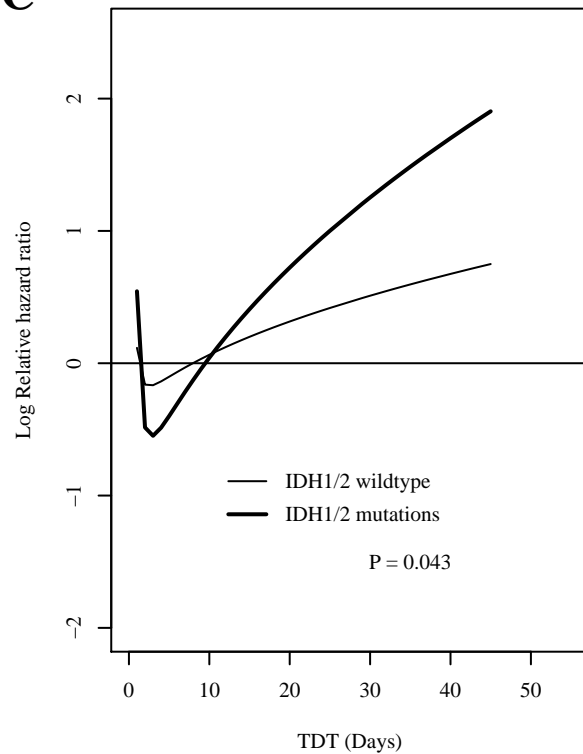

Supplement: S2 Fig — Log hazard in mortality for AML patients by IDH1/2 mutation status after adjusting clinical variables such as age (continuous), WBC (continuous) (A). Log hazard in mortality for AML patients by IDH1/2 mutation status after adjusting molecular variables like cytogenetic subtypes and genes mutations of FLT3ITD, CEBPA, DNMT3a, NPM1 and IDH1/2 in MFPIgen models(B). Log hazard in mortality for AML patients by IDH1/2 mutation status after adjusting clinical and molecular variables(C). (PDF) [file pone.0140622.s002.pdf]

**A****Age**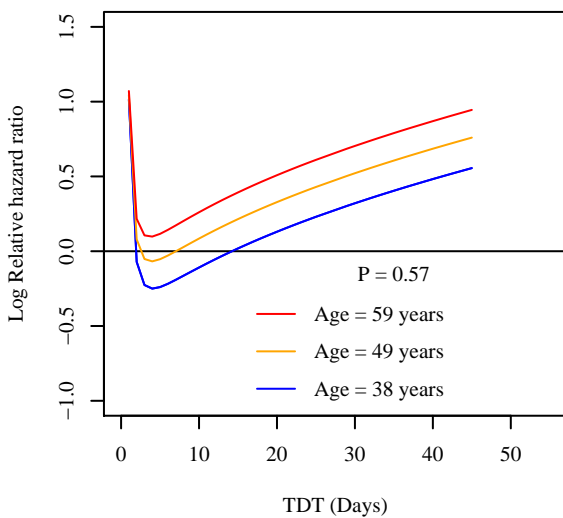**B****WBC**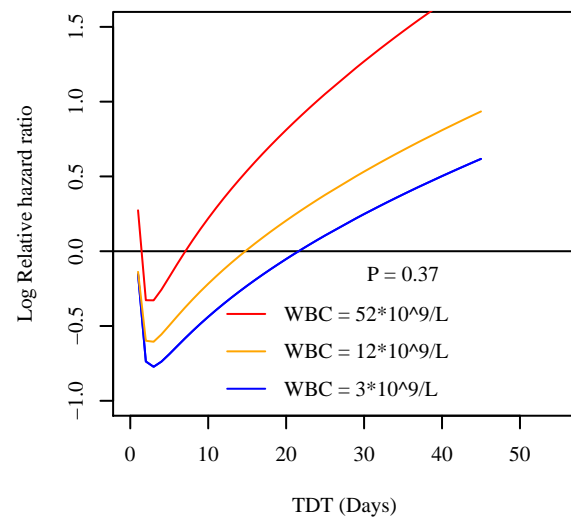**C****Blast**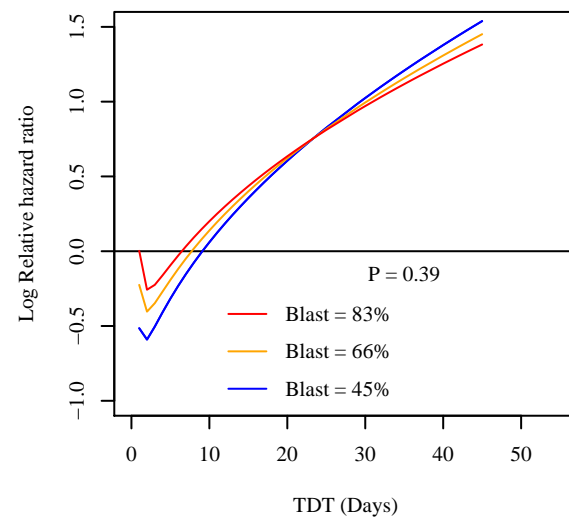**D****Cytogenetic subtype**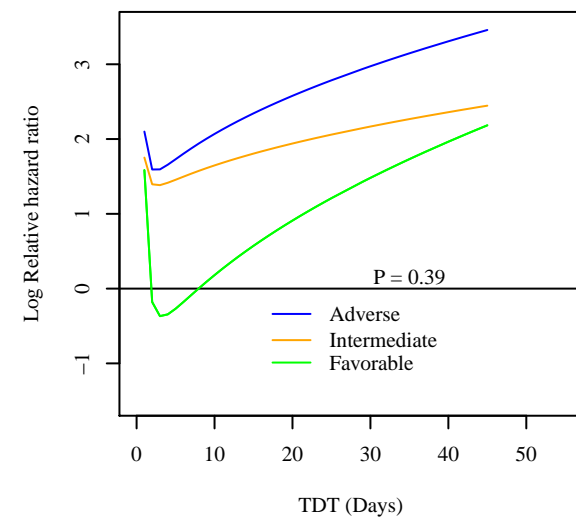**E****FLT3ITD**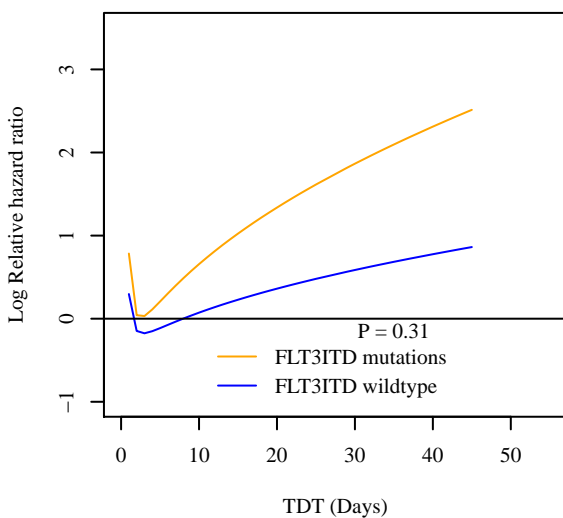**F****NPM1**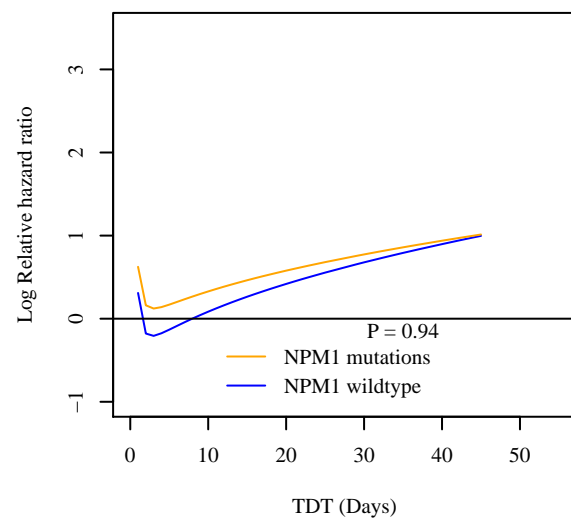**G****CEBPA**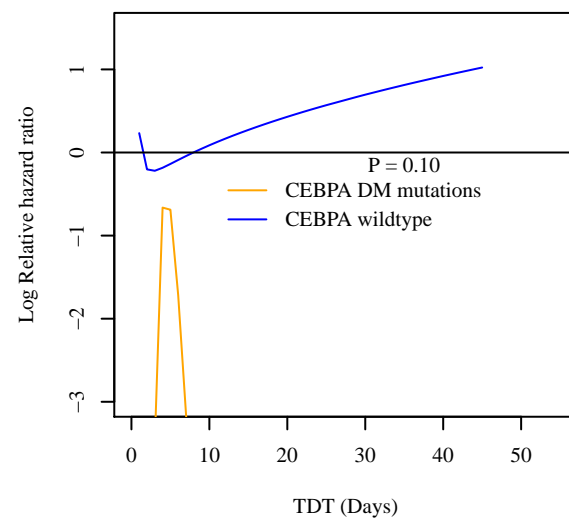**H****DNMT3a**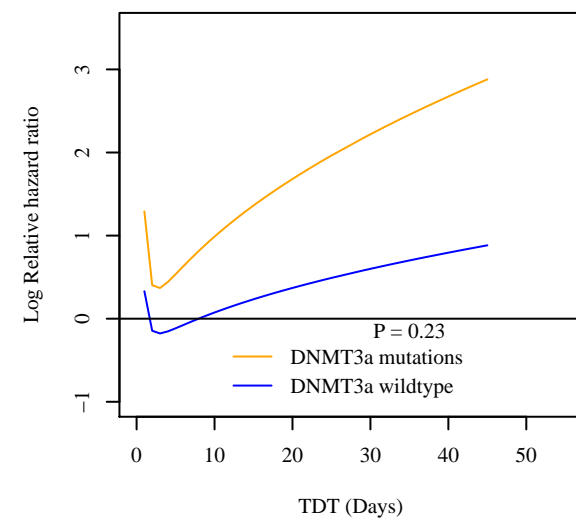

Supplement: S3 Fig — There were no interactive effect between TDT and covariates including age (A), WBC (B), percent blast (C), cytogenetic subtypes (D), and genes mutation of FLT3ITD (E), NPM1 (F), CEBPA (G) and DNMT3a (H). MFPIgen functions were used to estimate the significance of interaction between the predictors and TDT using fractional polynomial transformation with the powers (-2,0.5). (PDF) [file pone.0140622.s003.pdf]
