# Supplementary material for: Impact of Chemotherapy Delay on Overall Survival for AML with IDH1/2 Mutations: A Study in Adult Chinese Patients
Source: PLoS One. 2015 Oct 14;10(10):e0140622. doi: 10.1371/journal.pone.0140622 (PMC4605653; doi:10.1371/journal.pone.0140622)
Supplement: S1 Table — (DOCX) [file pone.0140622.s004.docx]

**S1Table.Univariate analysis for overall survival in AML patients.**

| **Variables** | **HR (95%CI)** | **P value** |
| --- | --- | --- |
| **Age,years** | 1.02(1.01,1.03) | 0.002 |
| **Percent blast** | 1.02(1.01,1.04) | 0.204 |
| **WBC** | 1.02(1.01,1.05) | 0.001 |
| **Cytogenetic risk group** |  |  |
| **Intermediate vs. Favorable** | 2.34(1.24,4.47) | 0.008 |
| **Adverse vs. Favorable** | 3.49(1.70,7.16) | 0.001 |
| ***FLT3*ITD** | 1.50(1.03,2.20) | 0.035 |
| ***CEBPA^DM^*** | 0.11(0.03,0.46) | <0.001 |
| ***NPM1*** | 1.24(0.9,1.71) | 0.190 |
| ***IDH1/2*** | 1.55(1.13,2.15) | 0.007 |
| ***DNMT3a*** | 1.96(1.32,2.93) | <0.001 |
| **TDT** |  |  |
| **Days 1—2 vs. Days 13—45** | 0.89(0.58,1.36) | 0.59 |
| **Days 3—4 vs. Days 13—45** | 0.55(0.33,0.91) | 0.02 |
| **Days 5—6 vs. Days 13—45** | 0.62(0.38,0.99) | 0.048 |
| **Days 7—8 vs. Days 13—45** | 0.72(0.43,1.23) | 0.24 |
| **Days 9—10 vs.Days 13—45** | 0.92(0.48,1.76) | 0.79 |
| **Days 11—12 vs. Days 13—45** | 1.25(0.55,2.81) | 0.59 |

WBC: white blood cell counts; DM: double-allele.
