# Supplementary material for: Impact of Chemotherapy Delay on Overall Survival for AML with IDH1/2 Mutations: A Study in Adult Chinese Patients
Source: PLoS One. 2015 Oct 14;10(10):e0140622. doi: 10.1371/journal.pone.0140622 (PMC4605653; doi:10.1371/journal.pone.0140622)
Supplement: S2 Table — (DOCX) [file pone.0140622.s005.docx]

**S2 Table. Multivariate analysis for overall survival in AML patients.**

| **Variables** | **HR (95% CI)** | **P value** |
| --- | --- | --- |
| ***IDH1/2*** | 1.09(0.77,1.54) | 0.623 |
| **TDT1** | 1.008(1.003,1.013) | 0.001 |
| **TDT2** | 2.71(1.78,4.12) | <0.001 |
| **WBC** | 1.004(1.002,1.005) | <0.001 |
| **Age** | 1.01(1.002,1.03) | 0.020 |
| **Cytogenetic risk group** |  |  |
| **Intermediate vs. Favorable** | 2.46(1.26,4.79) | 0.008 |
| **Adverse vs. Favorable** | 2.82(1.29,6.17) | 0.009 |
| ***FLT3*IDT** | 1.31(0.84,2.04) | 0.242 |
| ***CEBPA*^DM^** | 0.11(0.03,0.44) | 0.002 |
| ***NPM1*** | 0.89(0.62,1.30) | 0.557 |
| ***DNMT3a*** | 1.94(1.26,2.90) | 0.002 |

TDT1 and TDT2 denote the fractional polynomial terms TDTI^-2^ and TDTI^0.5^, where TDTI=TDT/10.WBC: white blood cell counts; DM: double-allele.
