# Supplementary material for: Impact of Chemotherapy Delay on Overall Survival for AML with IDH1/2 Mutations: A Study in Adult Chinese Patients
Source: PLoS One. 2015 Oct 14;10(10):e0140622. doi: 10.1371/journal.pone.0140622 (PMC4605653; doi:10.1371/journal.pone.0140622)
Supplement: S3 Table — (DOCX) [file pone.0140622.s006.docx]

**S3 Table. Distribution of FAB subgroup in patients with *IDH1/2* mutations by 7 days of delayed treatment.**

|  | **<7 days(n=41)** | **>=7 days(n=44)** | **P value** |
| --- | --- | --- | --- |
| **FAB classification** |  |  | 0.40 |
| **M0** | 7(17) | 11(25) |  |
| **M1** | 2(5) | 5(11) |  |
| **M2** | 16(39) | 13(30) |  |
| **M4** | 3(7) | 3(7) |  |
| **M5** | 10(24) | 12(27) |  |
| **M6** | 3(7) | 0(0) |  |

FAB: French-America-British.
