# Supplementary material for: Impact of Chemotherapy Delay on Overall Survival for AML with IDH1/2 Mutations: A Study in Adult Chinese Patients
Source: PLoS One. 2015 Oct 14;10(10):e0140622. doi: 10.1371/journal.pone.0140622 (PMC4605653; doi:10.1371/journal.pone.0140622)
Supplement: S4 Table — (DOCX) [file pone.0140622.s007.docx]

**S4 Table. Results of multivariate analysis comparing the interaction between TDT and *IDH1/2* mutations on overall survival.**

| **Variables** | **HR(95% CI)** | **P value** |
| --- | --- | --- |
| **TDT-by-IDH1/2** |  | 0.043 |
| **I(TDT1,IDH1/2)** | 1.01(1.002,1.02) | 0.046 |
| **I(TDT2,IDH1/2)** | 2.89(1.22,6.82) | 0.016 |
| **TDT1** | 1.005(0.999,1.01) | 0.092 |
| **TDT2** | 1.85(1.06,3.24) | 0.031 |
| ***IDH1/2*** | 0.88(0.57,1.34) | 0.547 |
| **WBC** | 1.003(1.001,1.01) | <0.001 |
| **Age** | 1.01(1.003,1.03) | 0.013 |
| **Cytogenetic risk group** |  |  |
| **Intermediate vs. Favorable** | 2.27(1.16,4.43) | 0.017 |
| **Adverse vs. Favorable** | 2.63(1.2,5.77) | 0.015 |
| ***FLT3*IDT** | 1.29(0.82,2.01) | 0.268 |
| ***CEBPA^DM^*** | 0.11(0.03,0.45) | 0.002 |
| ***NPM1*** | 0.91(0.63,1.31) | 0.608 |
| ***DNMT3a*** | 1.78(1.17,2.72) | 0.007 |

TDT-by-IDH1/2: interaction between TDT and IDH1/2 mutations. TDT1 and TDT2 denote the fractional polynomial terms TDTI^-2^ and TDTI^0.5^, where TDTI=TDT/10. I(TDT1,IDH1/2) and I(TDT2,IDH1/2) denotes the interactive term of TDT1-by-IDH1/2 and TDT2-by-IDH1/2, respectively. MFPIgen was used to estimate the interaction between TDT and IDH1/2 mutations in the different adjusted models. WBC: white blood cell counts. DM: double-allele.
